# Therapy-related AML: long-term outcome in a large cohort of AML-patients with intensive and non-intensive therapy

**DOI:** 10.1038/s41408-024-01140-5

**Published:** 2024-09-16

**Authors:** Sophia Gross, Jana Ihlow, Leonie Busack, Kacper Adamiak, Jens Schrezenmeier, Julia Jesse, Michaela Schwarz, Anne Flörcken, Lam Giang Vuong, Kathrin Rieger, Jan Krönke, Philipp le Coutre, Vivien Boldt, Ann-Christin von Brünneck, David Horst, Thomas Burmeister, Igor-Wolfgang Blau, Ulrich Keller, Lars Bullinger, Jörg Westermann

**Affiliations:** 1 Department of Hematology, Oncology and Tumor Immunology, Campus Virchow Clinic, Campus Charité-Mitte and Campus Benjamin Franklin, Charité – Universitätsmedizin Berlin, corporate member of Freie Universität Berlin and Humboldt-Universität zu Berlin, Charitéplatz 1, 10117 Berlin, Germany; 2 Institute of Pathology, Charité – Universitätsmedizin Berlin, corporate member of Freie Universität Berlin and Humboldt-Universität zu Berlin, Charitéplatz 1, 10117 Berlin, Germany; 3 Berlin Institute of Health at Charité – Universitätsmedizin Berlin, BIH Academy, BIH Charité Clinician Scientist Program, Charitéplatz 1, 10117 Berlin, Germany; 4 Charité-Universitätsmedizin Berlin, corporate member of Freie Universität Berlin and Humboldt-Universität zu Berlin, German Cancer Consortium (DKTK), partner site, Berlin, Germany; 5 Labor Berlin - Charité Vivantes GmbH, Berlin, Germany

**Keywords:** Epidemiology, Medical research

## Abstract

Therapy-related acute myeloid leukemia (t-AML) often exhibits adverse (genetic) features. There is ongoing discussion on the impact of t-AML on long-term outcome in AML. Therefore, we retrospectively analyzed clinical and biological characteristics of 1133 AML patients (225 t-AML patients and 908 de novo AML patients) with a median follow-up of 81.8 months. T-AML patients showed more adverse genetic alterations, higher age and more comorbidities as compared to de novo AML. Median OS in intensively treated t-AML patients was 13.7 months as compared to 39.4 months in de novo AML (*p* < 0.001). With non-intensive therapy, OS did not differ significantly (*p* = 0.394). With intensive therapy, significant differences in favor of de novo AML were observed in the ELN intermediate I/II (*p* = 0.009) and adverse (*p* = 0.016) risk groups but not within favorable risk groups (APL *p* = 0.927, ELN favorable *p* = 0.714). However, t-AML was no independent risk factor for OS (*p* = 0.103), RR (*p* = 0.982) and NRM (*p* = 0.320) in the multivariate analysis. A limitation of our study is an ELN 2010 risk stratification due to a lack of more comprehensive molecular data according to ELN 2022. We conclude that therapeutic algorithms in t-AML, in particular with regard to allo-HSCT, should be guided by ELN genetic risk rather than classification as t-AML alone. Our data support the WHO and ICC 2022 classifications, which include t-AML as diagnostic qualifier rather than a separate subcategory.

## Introduction

Therapy-related AML (t-AML) is a myeloid neoplasm that evolves secondary to cytotoxic therapy (chemotherapy and/or radiotherapy) for malignant or non-malignant diseases due to DNA damage in hematopoietic progenitor cells. Approximately 5–15% of adult AML patients are reported to have t-AML [1–4]. Chemotherapy and/or radiotherapy may cause mutations, leading to clonal hematopoiesis with subsequent selection of resistant clones during AML therapy. Especially alkylating agents and topoisomerase-II-inhibitors have been associated with mutagenic effects, typically with different latency periods [5, 6]. Over the past decades, increasing numbers of multidrug combinations in cancer therapy and improved long-term survival in cancer patients have led to an increasing incidence of t-AML [1, 7–10]. Owing to its secondary nature, t-AML is frequently associated with molecular aberrations and clonal complexity at baseline, adverse cytogenetics and preceding therapy-induced sequelae, which altogether may result in a poorer outcome as compared to de novo AML [1, 2, 11–17]. In the literature, there are partially conflicting results with regard to the role of t-AML as an independent prognostic factor. However, many studies are small or performed within particular subgroups only. Higher age and adverse genetics in t-AML can be interpreted as being secondary features in t-AML that may fully explain inferior survival. On the other hand, confounding variables such as preceding therapies with associated toxicities and possibly yet unknown t-AML-specific genetic features might cause increased comorbidity and resistance to therapy and thus lead to impaired overall survival. This aspect seems to be particularly relevant for patients, who are eligible for intensive therapy including allogeneic hematopoietic stem cell transplantation (allo-HSCT). In the current WHO and ICC classifications published in 2022, t-AML is included as a „diagnostic qualifier“ in addition to genetically and morphologically defined subgroups of AML (i.e. “AML with KMT2A mutation post cytotoxic therapy”) [18, 19]. However, there is an ongoing discussion as to whether t-AML per se is associated with an inferior clinical outcome and whether there are particular clinical or biological factors that determine the prognosis in t-AML [1, 2, 13, 14, 20–22]. To address these questions, we performed a large retrospective study in 1133 AML patients with the aim to compare clinical and biological baseline characteristics as well as OS, NRM and RR in t-AML versus de novo AML and to identify further risk factors in t-AML.

## Patients and methods

### Study design and endpoints

**Fig. 1:**
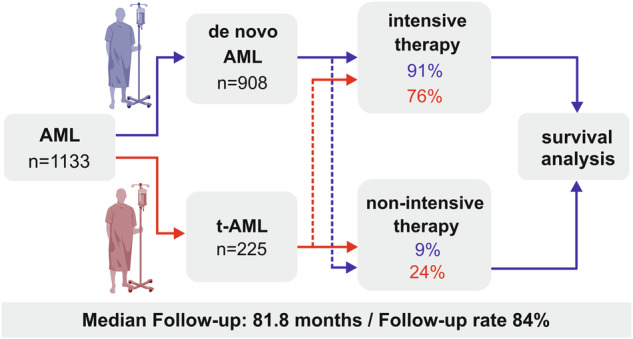
Study design. n number of patients, t-AML therapy-related AML.

1133 patients aged ≥18 years with newly diagnosed AML were treated at our center within the past two decades (January 1^st^, 1995–June 30th, 2018) and were eligible for this retrospective, non-interventional study. In our database, 20% (*n* = 225) had t-AML and 80% (*n* = 908) had de novo AML. T-AML was defined as secondary to previous cytotoxic therapy for solid cancer, hematologic malignancy or autoimmune disease [18, 19, 23]. In both groups, baseline characteristics, risk factors, remission rates, OS, RR and NRM were analyzed and compared between t-AML and de novo AML. OS, RR and NRM were defined as clinical endpoints by applying the Cheson criteria [24, 25] and the response criteria of the European Society for Blood and Bone Marrow Transplantation. Patients were further stratified with regard to type of administered therapy (intensive vs. non-intensive) and age (<60 years vs. ≥60 years). In patients with intensive therapy, cytarabine- and daunorubicin-based chemotherapy was administered as first-line induction therapy. Subsequently, patients received cytarabine- +/- anthracycline-based consolidation therapy and/or allo-HSCT following either myeloablative or reduced intensity conditioning regimens. Non-intensive therapy consisted of best supportive care +/- oral or parenteral chemotherapy (primarily low-dose AraC, hypomethylating agents or hydroxyurea). None of the patients received CPX-351 and only two patients were treated with Venetoclax since these drugs had not been approved during the study period. The study design is depicted in Fig. 1. Bone marrow evaluation was conducted using cytology, flow cytometry, histology and immunohistochemistry. The 2010 European LeukemiaNet (ELN) classification was applied for the assessment of the remission status and patients were stratified accordingly. ELN intermediate risk group I and II were merged as “ELN intermediate I/II” since previous studies had shown a lack of discriminatory power between these risk groups, particularly in older patients [26]. According to ELN, CR was defined as complete remission with hematological recovery, CRi was defined as complete remission with incomplete hematological recovery and MLFS was referred to as morphologic leukemia free state [3]. For the analysis, CR, CRi and MLFS were summarized as overall response rate (ORR). Partial remission (PR) was defined by a decrease of bone marrow blasts by at least 50% to a blast percentage in the range of 5–25%. Primary refractory disease (RD) was defined as a lack of CR or CRi after two courses of intensive induction treatment [18]. Furthermore, patients were stratified with regard to their molecular/cytogenetic risk group according to the ELN 2010 classification [27], due to a lack of additional molecular data that are mandatory for the ELN 2022 classification. The patients’ general condition was measured by the Eastern Cooperative Oncology Group-performance score (ECOG) [28]. Comorbidity was assessed using the Charlson Comorbidity Index (CCI) [29]. The study was in line with the Declaration of Helsinki and was approved by the local ethics committee (EA4/026/23).

### Statistical analysis

Data curation and retrospective analysis were performed using SPSS Statistics, version 25 (IBM^®^, 2022, Armonk, NY). Baseline characteristics were analyzed using non-parametric tests, such as the Mann-Whitney U test in two subgroups, the Kruskal-Wallis-H-test in multiple subgroups or the Chi-Square test for nominal variables followed by post-hoc testing and Bonferroni adjustment [30]. Median follow-up was analyzed by using the reverse Kaplan–Meier method [31]. OS was analyzed using the Kaplan-Meier method. For comparisons in OS, the logrank test was applied. To define a hazard-ratio (HR), a univariate Cox regression model was applied. To determine the HR of independent risk factors, a multivariate Cox proportional hazards model was used. The latter included factors with a univariate significance level of *p* < 0.05. In order to define a hazard ratio (HR), some variables were transformed into categorical dichotomous data. To estimate the risk of relapse and non-relapse mortality, a multivariate cause-specific Cox proportional hazards model that included confounding factors with significant impact on relapse and survival was used based on an etiological approach. Within this model, death and relapse were defined as competing events and treated as censored observations [32]. In order to address possible multicollinearity between t-AML and ELN adverse risk that could have concealed the impact of t-AML on OS, subgroup analyses were performed within the different ELN risk groups. Furthermore, variance inflation factor (VIF) analysis for multicollinearity was performed for all factors included in the multivariate Cox regression model using survival as dependent variable. VIF-values of 1-2 were interpreted as lack of relevant collinearity, VIF-values > 2 and ≤ 4 were interpreted as mild collinearity, VIF-values > 4 should have warranted further investigation and VIF values ≥ 10 would have meant severe collinearity with subsequent exclusion of the factor which was not the case in our study (https://cran.r-project.org/web/packages/olsrr/vignettes/regression_diagnostics.html). A p-value of *p* < 0.05 was considered statistically significant. For graphical presentation, Graph Pad Prism 8 (GraphPad Software.Inc), Corel Draw Graphics Suite (Version 22, 2020) and BioRender.com (Fig. 1) were applied.

## Results

### Adverse baseline characteristics accumulate in t-AML

**Fig. 2:**
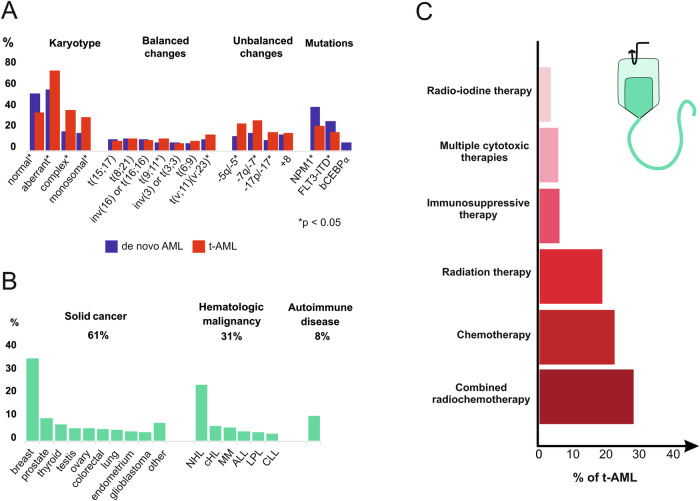
Baseline characteristics in t-AML-patients. **A** Karyotype and genetic alterations at baseline in t-AML vs. de novo AML. **B** Distribution of the most frequent primary diseases in the t-AML cohort, **C** Types of therapies that had been applied prior to primary diagnosis of t-AML. t translocation, *inv* inversion, NHL Non-Hodgkin lymphoma, cHL classical Hodgkin lymphoma, MM multiple myeloma, ALL acute lymphoblastic leukemia, LPL lymphoplasmacytic lymphoma, CLL chronic lymphocytic leukemia.

In the entire cohort (*n* = 1133), median follow up was 81.8 months. Of all patients included in this study, 20% had t-AML (*n* = 225) and 80% (*n* = 908) were diagnosed with de novo AML. 91% of de novo AML patients (*n* = 823/908) and 76% (*n* = 172/225) of t-AML patients were eligible for intensive therapy (*p* < 0.001). T-AML patients were more likely to be female, were significantly older and had more comorbidities as well as a higher frequency of unfavorable cytogenetic risk factors as compared to de novo AML patients, such as complex karyotypes, monosomal karyotypes and unbalanced translocations (Table 1, Fig. 2A). These differences led to a doubling of patients with adverse ELN 2010 risk features in our t-AML cohort. Median latency period from initial diagnosis to t-AML was 69.2 months. Of all t-AML patients, 61% (*n* = 138/225) had been diagnosed with solid cancer prior to development of AML with highest numbers for breast cancer (*n* = 74/225) and prostate cancer (*n* = 16/225). 31% of t-AML patients (*n* = 69/225) had hematological malignancies and 8% (*n* = 18/225) were diagnosed with an autoimmune disease (Fig. 2B). Active cancer was present in 16% of t-AML patients (*n* = 37/225). Combined radiochemotherapy had been administered in 33% of t-AML patients (*n* = 75/225), which was followed by chemotherapy and radiation therapy alone (Fig. 2C). For autoimmune diseases, patients had received either azathioprine, mitoxantrone, cyclophosphamide or methotrexate (MTX). MTX monotherapy had been administered in a few patients only (*n* = 8/225). Antecedent t-MDS was present in 25% of t-AML patients (*n* = 57/225). A detailed characterization of previous diseases and therapies is shown in Fig. 2 and Table 2.

**Table 1:** Baseline characteristics in 1133 patients with t-AML vs. de novo AML.

Characteristics	Entire cohort	t-AML	de-novo AML	*p* value
*n* (%)	1133	225 (20)	908 (80)	–
Eligible for intensive therapy, *n* (%)	995 (88)	172 (76)	823 (91)	**<0.001**
Sex (female), *n* (%)	570 [50]	144 [64]	426 [47]	**<0.001**
- intensive therapy	504 [51]	116 [67]	388 [47]	**<0.001**
- non-intensive therapy	66 [48]	28 [53]	38 [45]	0.451
Age, years, median [IQR]	56 [45–66]	61 [52–69]	55 [43–65]	**<0.001**
- intensive therapy	54 [43–63]	57.5 [49–66]	53 [42–62]	**<0.001**
- non-intensive therapy	74 [67–78]	72 [65–79]	74 [69–78]	0.087
ECOG-PS, median [IQR]	1 [0-1]	1 [0-1]	1 [0-1]	0.075
- intensive therapy	1 [0-1]	1 [0-1]	1 [0-1]	0.524
- non-intensive therapy	1 [1-2]	1 [1-2]	1.5 [1-2]	0.123
CCI, median [IQR]	0 [0-2]	2 [2-3]	0 [0-1]	**<0.001**
- intensive therapy	0 [0-2]	2 [2-3]	0 [0-1]	**<0.001**
- non-intensive therapy	2 [1–3]	3 [2–4]	1 [0–3]	**<0.001**
Hemoglobin, g/dl, median [IQR]	9.1 [7.7–10.2]	8.9 [8.0–10.1]	9.1 [7.6–10.3]	0.781
- intensive therapy	9.1 [7.6–10.3]	8.9 [7.9–10.2]	9.1 [7.5–10.3]	0.978
- non-intensive therapy	9 [8.1–10.0]	9.3 [8.3–10.1]	9.0 [8.1–9.9]	0.523
WBC, count/nl, median [IQR]	9.7 [2.6–45.8]	4.7 [2.0–24.3]	12.5 [2.9–51.5]	**<0.001**
- intensive therapy	8.8 [2.6–45.2]	4.4 [1.9–20.6]	11.5 [2.8–50.9]	**<0.001**
- non-intensive therapy	13.3 [2.9–51.2]	5.2 [2.2–34.8]	22.7 [5.4–65.7]	**0.003**
PLT, count/nl, median [IQR]	55 [28–106.7]	53.5 [24.0–94.5]	56.0 [30–108]	0.272
- intensive therapy	55.0 [28–108]	53.5 [25.3–93.5]	56.0 [29.0–108.5]	0.560
- non-intensive therapy	57.0 [30.0–97.2]	56.5 [18–96.3]	57.0 [33.0–98.0]	0.264

*n* number of patients, *IQR* interquartile range, *ECOG-PS* Eastern cooperative oncology group performance score, *CCI* Charlson Comorbidity Index, *ELN* European LeukemiaNet, *ECOG* Eastern cooperative oncology group.

**Table 2:** Primary diseases in 225 t-AML patients grouped for intensive and non-intensive therapy.

Entity	Entire t-AML cohort	Intensive therapy	Non-intensive therapy	*p* value
***n*** **[%]**	**225 [100]**	**172 [76]**	**53** [14]	
**Solid cancer,** ***n*** **[%]**	**138 [61]**	**99 [58]**	**39 [74]**	**0.036**
Breast carcinoma	74 [33]	60 [35]	14 [26]	
Prostate carcinoma	16 [7]	9 [5]	7 [13]	
Thyroid carcinoma	10 [4]	7 [4]	3 [6]	
Tumor of testicles	6 [3]	5 [3]	1 [2]	
Ovarian carcinoma	6 [3]	4 [2]	2 [4]	
Colorectal carcinoma	5 [2]	2 [1]	3 [6]	
Lung carcinoma	4 [2]	1 [0.5]	3 [6]	
Endometrial carcinoma	3 [1]	3 [2]	─	
Glioblastoma	2 [1]	1 [0.5]	1 [2]	
Neuroendocrine tumor	2 [1]	1 [0.5]	1 [2]	
Uterine sarcoma	2 [1]	2 [1]	─	
Ewing Sarcoma	1 [0.5]	1 [0.5]	─	
Liposarcoma	1 [0.5]	1 [0.5]	─	
Melanoma	1 [0.5]	1 [0.5]	─	
Nasopharyngeal carcinoma	1 [0.5]	─	1 [2]	
Osteosarcoma	1 [0.5]	1 [0.5]	─	
Squamous cell carcinoma of the skin	1 [0.5]	─	1 [2]	
Squamous cell carcinoma of the tonsil	1 [0.5]	─	1 [2]	
Carcinoma of the cervix	1 [0.5]	─	1 [2]	
**Hematologic malignancies,** ***n*** **[%]**	**69** [31]	**58** [34]	**11** [21]	0.073
B-cell Non-Hodgkin lymphoma	48 [21]	42 [24]	6 [11]	
Classical Hodgkin Lymphoma	8 [4]	8 [5]	─	
Multiple Myeloma	7 [3]	4 [2]	3 [6]	
Acute lymphoblastic leukemia	3 [1]	3 [2]	─	
Lymphoplasmacytic lymphoma	2 [1]	1 [0.5]	1 [2]	
Chronic lymphocytic leukemia	1 [0.5]	─	1 [2]	
**Autoimmune disease,** ***n*** **[%]**	**18** [8]	**15** [9]	**3** [6]	0.473
Rheumatoid arthritis	6 [3]	5 [3]	1 [2]	
Multiple sclerosis	3 [1]	3 [2]	─	
Psoriasis	2 [1]	2 [1]	─	
Autoimmune hemolytic anemia	1 [0.5]	1 [0.5]	─	
Other autoimmune diseases	6 [3]	4 [2]	2 [4]	

### Overall survival in t-AML subgroups: treatment modality and age

**Fig. 3:**
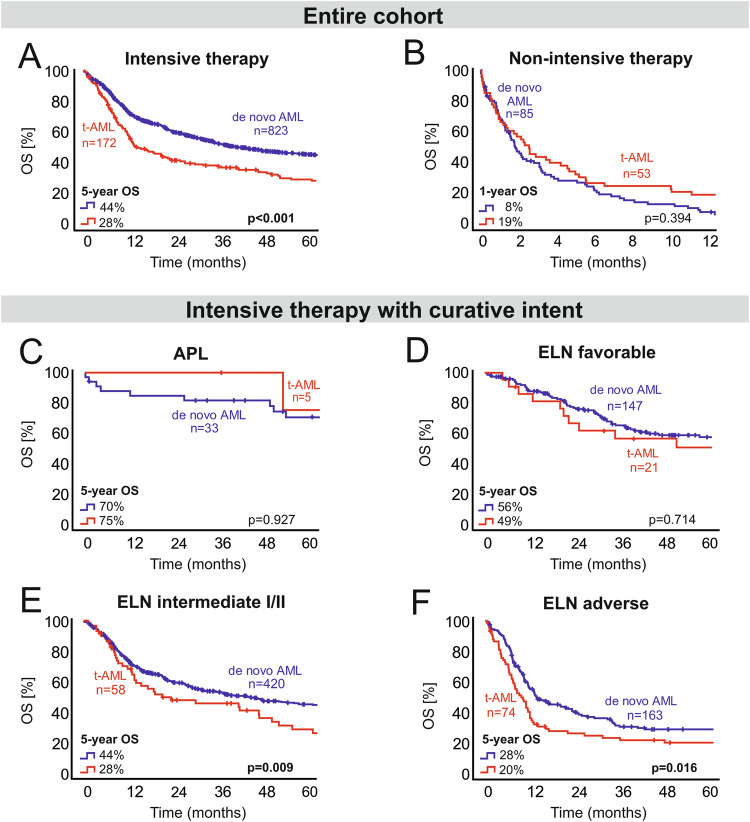
OS in t-AML vs. de novo AML (logrank test). **A** OS within the entire cohort of intensively treated patients: survival with t-AML is inferior to de novo AML. **B** OS within the entire cohort of non-intensively treated patients: no significant difference between t-AML and de novo AML. **C** OS of patients with APL: no significant difference between t-APL and de novo APL. **D** OS of patients with ELN 2010 favorable risk: no significant difference between t-AML and de novo AML. **E** OS of patients with ELN 2010 intermediate risk: survival with t-AML is inferior to de novo AML. **F** OS of patients with ELN 2010 adverse risk: survival with t-AML is inferior to de novo AML. n number of patients, OS overall survival, t-AML therapy-related AML, APL acute promyelocytic leukemia, ELN European LeukemiaNet.

Median OS was 24 months in the entire cohort (95% CI 19.5–28.5) and 5-year OS was 36.6% respectively. In intensively treated patients, OS was significantly inferior (*p* < 0.001) in t-AML as compared to de novo AML (5-year OS 28% vs. 44%, median OS 13.7 months vs. 39.4 months, Fig. 3A). In patients with non-intensive therapy, OS was particularly poor in the vast majority of patients, both with t-AML and de novo AML. In this subgroup, 1-year OS was 19% in t-AML vs. 8% in de novo AML. Median OS was 2.6 months in t-AML vs. 1.8 months in de novo AML, respectively (*p* = 0.394, Fig. 3B). In patients with intensive therapy aged < 60 years, the inferior OS of t-AML was maintained. Comparing t-AML (*n* = 96/225) and de novo AML (*n* = 562/908) in this age group, we found that 5-year OS and median OS were 31% and 19.3 months in t-AML vs. 51% and 64.6 months in de novo AML (*p* < 0.001). In contrast, patients aged ≥ 60 years showed 5-year OS rates of 23% in t-AML vs. 30% in de novo AML with a median OS of 12.4 months in t-AML vs. 23.3 months in de novo AML (*p* = 0.066).

### ELN risk groups and overall survival in t-AML

To address the impact of genetic risk groups on the outcome of t-AML with intensive therapy, survival analysis was performed within the different risk groups according to ELN 2010 (Fig. 3C–F). Within the ELN intermediate I/II and adverse risk groups, survival of patients with t-AML was inferior to patients with de novo AML. In the ELN intermediate I/II risk group, 5-year OS was 28% with t-AML vs. 44% with de novo AML (median OS 21.3 vs. 41.3 months, *p* = 0.009). In the ELN adverse risk group, 5-year OS was 20% with t-AML vs. 28% with de novo AML (median OS 10.1 vs. 14.3 months, *p* = 0.016). In both of these ELN risk groups, t-AML patients were significantly older than patients with de novo AML. Median age was 60 vs. 53 years in ELN intermediate I/II risk patients (*p* = 0.001) and 58 vs. 55 years in the adverse ELN risk group (*p* = 0.029). Furthermore, t-AML patients had a tendency towards a lower ORR after induction therapy, so that standard consolidation therapy was less frequently completed. In ELN intermediate I/II risk patients, ORR was 73% with t-AML and 84% with de novo AML (*p* = 0.094). In ELN adverse patients, ORR was 63% in t-AML vs. 73% in de novo AML (*p* = 0.290). Within the ELN intermediate I/II risk group, consolidation chemotherapy was applied in 52% of patients with t-AML vs. 68% of patients with de novo AML. Within the ELN adverse risk group, only 35% of patients with t-AML received consolidation chemotherapy as compared to 50% in de novo AML. In contrast, there were no significant differences in OS between patients with t-AML and de novo AML in both the ELN favorable risk group (*p* = 0.714) and in acute promyelocytic leukemia (APL, *p* = 0.927). In the ELN favorable risk group, 5-year OS was 49% in t-AML vs. 56% in de novo AML (median OS 53.5 vs. 168.4 months, *p* = 0.714). In patients with t-APL, 5-year OS was 75 vs. 70% in de novo APL (median OS 107.9 vs. 198.3 months, *p* = 0.927). Patients within the ELN favorable risk group showed well balanced baseline characteristics, including age with a median age of 54 years in t-AML vs. 52 years in de novo AML (*p* = 0.878). Additionally, ORR and consolidation therapy rates were comparable in ELN favorable risk patients and patients with APL. In the ELN favorable risk group, ORR was 95% in t-AML vs. 92% in de novo AML (*p* = 1.000). In APL patients, ORR was 80% in t-APL vs. 94% in de novo APL (*p* = 0.362).

In first remission (after induction therapy), 38% of de novo AML patients and 37% of t-AML patients received allo-HSCT (*p* = 0.887). There was no significant difference in transplantation rates between t-AML and de novo AML within the different genetic risk groups. In the entire cohort, OS of t-AML patients who received allo-HSCT in first remission was significantly worse than OS of de novo AML patients (median OS 21.0 vs. 90.8 months; 5-years OS 39 vs. 55%, *p* = 0.016). This was accompanied by an accumulation of ELN adverse risk patients in the t-AML cohort (54% of t-AML patients vs 29% in de novo AML). Considering long-term outcome of patients transplanted in first remission, OS in t-AML was comparable to de novo AML within the different ELN risk groups (ELN favorable *p* = 0.196, ELN intermediate I/II *p* = 0.178, ELN adverse *p* = 0.510). In an additional survival analysis within the favorable ELN risk group (t-AML vs. de novo AML), patients receiving allo-HSCT in first remission were censored at the time point of allo-HSCT in order to exclude the influence of a possibly different transplantation strategy in this subgroup. In this additional analysis, the lack of difference in OS within the favorable ELN risk group was maintained (*p* = 0.745).

### T-AML: specific clinical risk factors for overall survival

**Fig. 4:**
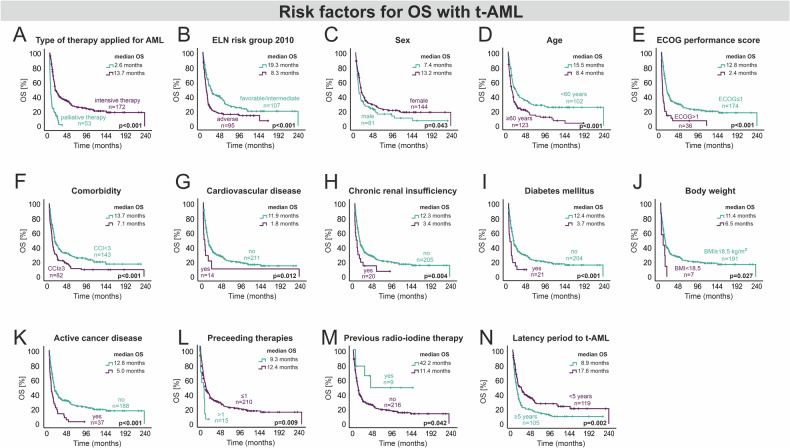
Baseline characteristics of t-AML patients with significant impact on OS (logrank-test). **A** Type of AML therapy, **B** ELN adverse risk, **C** sex differences, **D** age-related survival difference, **E** ECOG performance score, **F** comorbidity, **G** cardiovascular disease, **H** chronic renal insufficiency, **I** diabetes mellitus, **J** body weight, **K** active cancer disease, **L** preceeding therapies, **M** previous radio-iodine therapy, **N** Latency period to t-AML. *n* Number of patients, OS overall survival, t-AML therapy-related AML, ELN European LeukemiaNet, ECOG Eastern Cooperative Oncology Group, CCI Charlson comorbidity index, BMI body mass index.

**Fig. 5:**
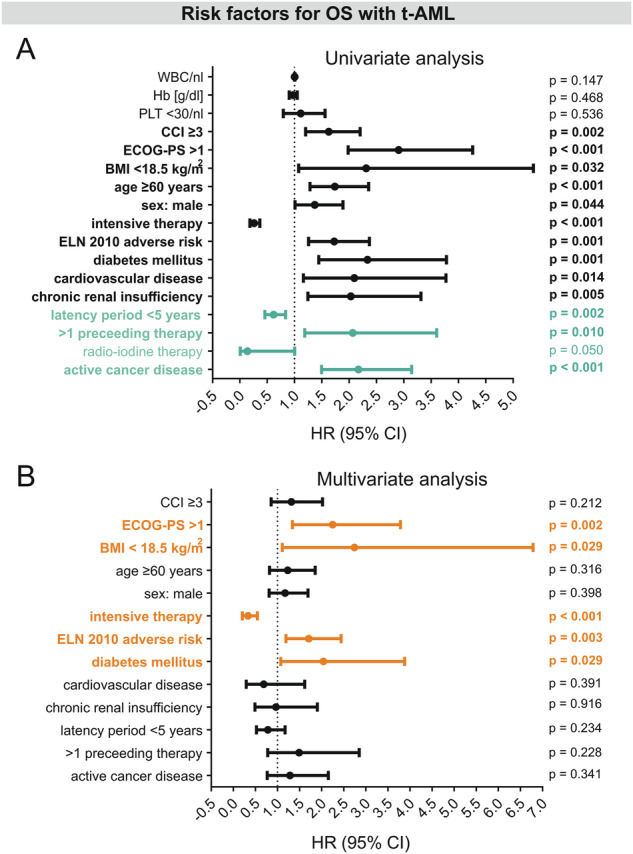
Risk factors for overall survival (OS) in 225 patients with t-AML. **A** Univariate Cox regression shows hazard ratio defined as risk of death from several risk factors in t-AML. Factors that were mainly determined by the previous disease are depicted in turquoise letters. **B** Multivariate Cox regression shows HR defined as risk of death from factors with univariate significance in t-AML and reveals ECOG-PS > 1, an adverse cytogenetic/molecular risk according to the ELN 2010 classification, intensive therapy, BMI < 18.5 kg/m^2^ and diabetes mellitus as independent risk factors for OS in t-AML (depicted in orange letters). WBC white blood cell count, Hb hemoglobin, PLT platelets, CCI Charlson comorbidity index, ECOG Eastern Cooperative Oncology Group, BMI body-mass index, ELN European LeukemiaNet, HR hazard ratio, CI confidence interval.

In order to identify risk factors which may contribute to the poor outcome in t-AML, t-AML patients were thoroughly analyzed (Fig. 4). As expected, active cancer disease was associated with a higher risk of death (HR 2.2). More than one preceding cytotoxic therapy had an adverse impact on OS (HR 2.1). In contrast, patients with an antecedent radioiodine therapy had a particularly favorable outcome (HR 0.4). However, patient numbers within these subgroups were rather small. The type of underlying disease (solid cancer vs. hematological malignancy vs. autoimmune disease) did not affect the OS significantly (*p* = 0.580). With regard to baseline characteristics at initial diagnosis of AML, t-AML patients with an ECOG score >1 (HR 2.3), an adverse ELN 2010 risk (HR 1.7), non-intensive therapy (HR 3.0), underweight (HR 2.7) and diabetes mellitus (HR 2.0) were at higher risk of death as confirmed by the multivariate analysis (Fig. 5).

### T-AML as a risk factor

**Fig. 6:**
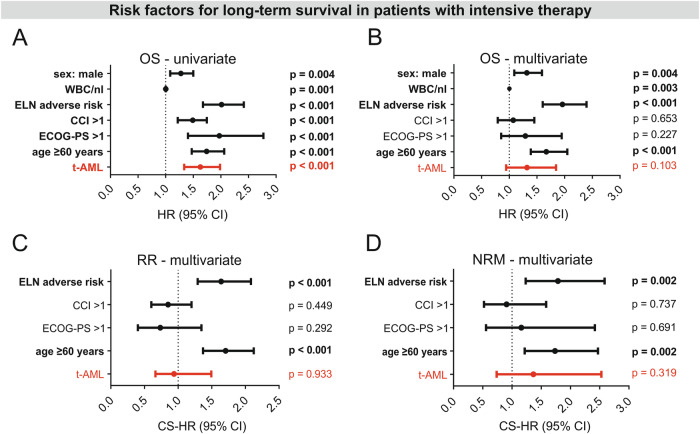
Risk factors for long-term survival in 995 AML patients with intensive therapy. **A** Univariate Cox regression. **B** Multivariate Cox regression. **C** Cause-specific hazard ratios for non-relapse mortality in the presence of risk factors with univariate impact. **D** Cause-specific hazard ratios for risk of relapse in the presence of risk factors with univariate impact. T-AML is no independent risk factor in the multivariate analysis of intensively treated patients, neither for OS, nor for RR or NRM. OS overall survival, RR risk of relapse, NRM non-relapse mortality, t-AML therapy-related AML, CCI Charlson comorbidity Index, ECOG Eastern Cooperative Oncology Group, ELN European LeukemiaNet, HR hazard ratio, CI confidence interval.

The important impact of baseline characteristics that was observed in the ELN subgroup analysis, was maintained in the multivariate analysis of patients with intensive therapy (Fig. 6). ELN 2010 adverse risk, higher age ($\ge$60 years), high leukocyte counts, and male sex could be confirmed as independent risk factors for OS. In contrast, t-AML itself was no independent risk factor in the entire cohort, neither for OS (*p* = 0.103, HR 1.3) nor for RR (*p* = 0.933, HR 0.9) or NRM (*p* = 0.319, HR 1.4). Potential collinearity between adverse genetic risk and t-AML which could have concealed t-AML as independent risk factor in the multivariate analysis of the entire cohort was addressed by 1) VIF-analysis for t-AML and ELN adverse risk and 2) multivariate analysis within the different ELN 2010 risk groups to avoid genetic risk as confounding cofactor for t-AML. VIF values for collinearity in the model including all factors with univariate significance for OS were 1.085 for ELN 2010 adverse risk and 2.217 for t-AML, respectively. Furthermore, VIF values were < 2.0 for all other covariates in the model (age > 60 years, male sex, ECOG-PS > 1, leukocyte count) except for CCI > 1 (VIF 2.196). This indicated minimal but no severe collinearity between t-AML and CCI but no relevant collinearity between the other parameters. Multivariate analysis within the ELN risk groups confirmed t-AML to be no independent risk factor for OS, RR and NRM, although there was borderline significance for inferior OS of t-AML patients within the intermediate I/II risk group (*p* = 0.055, Supplementary Fig. 1).

## Discussion

According to recent studies, t-AML has a frequency of up to 15% of all AML patients, with increasing incidence over the past decades [1, 3]. In our cohort, baseline characteristics, latency period and OS of t-AML patients were comparable to other studies [2, 9, 11, 22, 33]. In concordance with previous findings, the most frequent underlying malignancies were breast cancer and Non-Hodgkin lymphoma (NHL) in our cohort [2, 9, 19, 26, 34]. The high incidence of t-AML following breast cancer reflects the general population-based increase as well as advances in diagnosis and therapy, resulting in an improved long-term survival of breast cancer patients over the past decades [34, 35]. A possible explanation for the high percentage of t-AML following NHL might be frequent application of poly-chemotherapy together with increasing cure rates [35]. Preceding therapy-associated sequelae in t-AML may have an impact on comorbidities, which is mirrored by a higher CCI in our t-AML cohort.

The impact of t-AML as a risk factor has remained a matter of discussion over the past years due to partially conflicting results in different retrospective studies [1, 2, 13, 14, 20–23]. Our retrospective study shows that the inferior OS in t-AML mainly results from differences in clinical baseline characteristics and genetics. Although OS in t-AML is clearly inferior to de novo AML in general, long-term outcome of t-AML is comparable with de novo AML, if baseline characteristics and genetics are balanced. We hypothesize that the accumulation of adverse genetic and patient-associated risk factors in t-AML has an impact on long-term survival differences between t-AML and de novo AML patients. In our cohort, the differences in OS in favor of de novo AML reached beyond 15% after 5 years. This particularly affected patients who were younger than 60 years or were belonging to the ELN intermediate I/II and adverse risk groups that showed higher median age and signs of increased chemoresistance in terms of lower response rates. In these subgroups, it seems conceivable that inferior survival is mediated by additional genetic aberrations in t-AML that would have been detected by a more comprehensive molecular panel as proposed by the recent ELN 2022 recommendations, particularly within the intermediate I/II and adverse ELN risk groups [3]. Furthermore, “hidden” mutational and clonal complexity due to clonal selection over time is likely to affect OS in t-AML [12, 15–17, 36–39]. Interestingly, these differences resolved in intensively treated patients within the ELN favorable risk group and ─ in line with current literature ─ in APL patients [11, 40, 41] as well as in patients who were older than 60 years and had received non-intensive therapy. This may be explained by the fact that baseline characteristics in these subgroups were better balanced.

In this regard, several known risk factors with an adverse impact on OS in t-AML were confirmed in our multivariate analysis (ELN adverse risk, higher ECOG score and non-intensive therapy) [2, 11, 33, 42, 43]. In addition, underweight and diabetes were identified as independent risk factors in t-AML. Additionally, the univariate analysis showed an inferior OS in patients with more than one prior cytotoxic therapy and a trend towards better OS in patients with radio-iodine therapy. The latter group was mostly comprised of young and fit women who had been treated for thyroid cancer and thus were at risk of developing AML [2, 44–46]. According to the WHO, the role of radionucleotides in the pathogenesis of t-AML is unclear [18, 47] and our findings warrant further investigations in larger cohorts.

Despite the adverse outcome of t-AML patients as an entire group, t-AML per se was no independent risk factor, neither for OS nor for RR or NRM in intensively treated patients. Any difference in survival parameters between t-AML and de novo AML did not reach the level of significance in multivariate analysis although many adverse molecular features that are part of the ELN 2022 risk classification and are accumulated in t-AML were not available for our analysis. This lack of more comprehensive molecular data is a limitation of our study which is probably most relevant within the ELN 2010 intermediate I/II risk group. This subgroup comprises many AML patients with normal karyotype which would have been classified as “adverse risk” according to ELN 2022 due to additional molecular data including MDS-related abnormalities. Interestingly, in this subgroup, multivariate analysis showed borderline significance for t-AML (*p* = 0.055) as an independent prognostic factor (Supplemental Fig. 1). Nevertheless, we acknowledge that further, yet unknown factors might have an impact on survival in t-AML.

Besides classification and prognostication, the impact of t-AML becomes particularly important for therapeutic decision making in young/fit favorable risk t-AML patients, since the attribution of high-risk disease can lead to allo-HSCT in first CR in these patients. Instead, t-AML patients can be treated with consolidation chemotherapy when classified as favorable risk AML and showing MRD clearance at predefined timepoints [3]. Our study strongly suggests that ELN favorable risk t-AML patients (AML with NPM1^mut^ or core binding factor AML) as well as patients with t-APL are not at a higher risk for relapse or death as compared to de novo AML patients and thus should not routinely undergo allo-HSCT in first CR. Our findings are in accordance with a recent Swedish registry-based study also showing that t-AML is no independent risk factor in patients belonging to the favorable ELN 2010 risk group (with and without APL), whereas it is associated with a higher risk of death in ELN 2010 intermediate I/II and ELN 2010 adverse risk t-AML patients [1]. Moreover, another recently published retrospective study in NPM1^mut^ AML showed that NPM1^mut^ t-AML and de novo NPM1^mut^ AML have overlapping genetic features without differences in OS with regard to the multivariate analysis [48, 49].

Another clinically relevant question arising from these data is the most appropriate chemotherapy for t-AML patients within the favorable ELN risk group. On the one hand, a liposomal formulation of cytarabine and daunorubicin (CPX-351) has been shown to be superior to a standard cytarabine/anthracycline-based regimen in AML-MRC and t-AML. On the other hand, this survival advantage was predominantly demonstrated in a cohort of older AML patients aged > 60 years, containing only about 5% patients with favorable cytogenetics according to National comprehensive cancer network (NCCN) criteria and was most prominent within the subgroup that underwent allo-HSCT as consolidation treatment [50, 51]. Furthermore, consolidation chemotherapy in this trial was different from ELN standard recommendations since it did not contain repetitive cycles of intermediate dose cytarabine (ID AraC). Whether the outcome of consolidation therapy with CPX-351 is comparable or superior to ID AraC remains an open question, particularly in younger patients, and will assumingly be answered by currently active AML trials (AMLSG 30-18 trial/ NCT03897127) [52, 53].

In synopsis with current literature, our study strongly supports the view that risk stratification in t-AML, particularly with regard to the indication for allo-HSCT, should align with strategies used in de novo AML, both in intensive and non-intensive therapy. Additionally, our data support the current WHO and ICC 2022 classifications [18, 19], which consider t-AML as a “diagnostic qualifier” within the different AML subgroups rather than a separate subcategory.

## Supplementary information

Supplemental Figure 1

Supplemental Figure 1 Legend

Checklist

## Data Availability

The datasets generated during and/or analyzed during the current study are not publicly available due to ethical restrictions but are available from the corresponding author upon reasonable request.
