# Supplementary figures and images for: Therapy-related AML: long-term outcome in a large cohort of AML-patients with intensive and non-intensive therapy
Source: Blood Cancer J. 2024 Sep 16;14(1):160. doi: 10.1038/s41408-024-01140-5 (PMC11405931; doi:10.1038/s41408-024-01140-5)

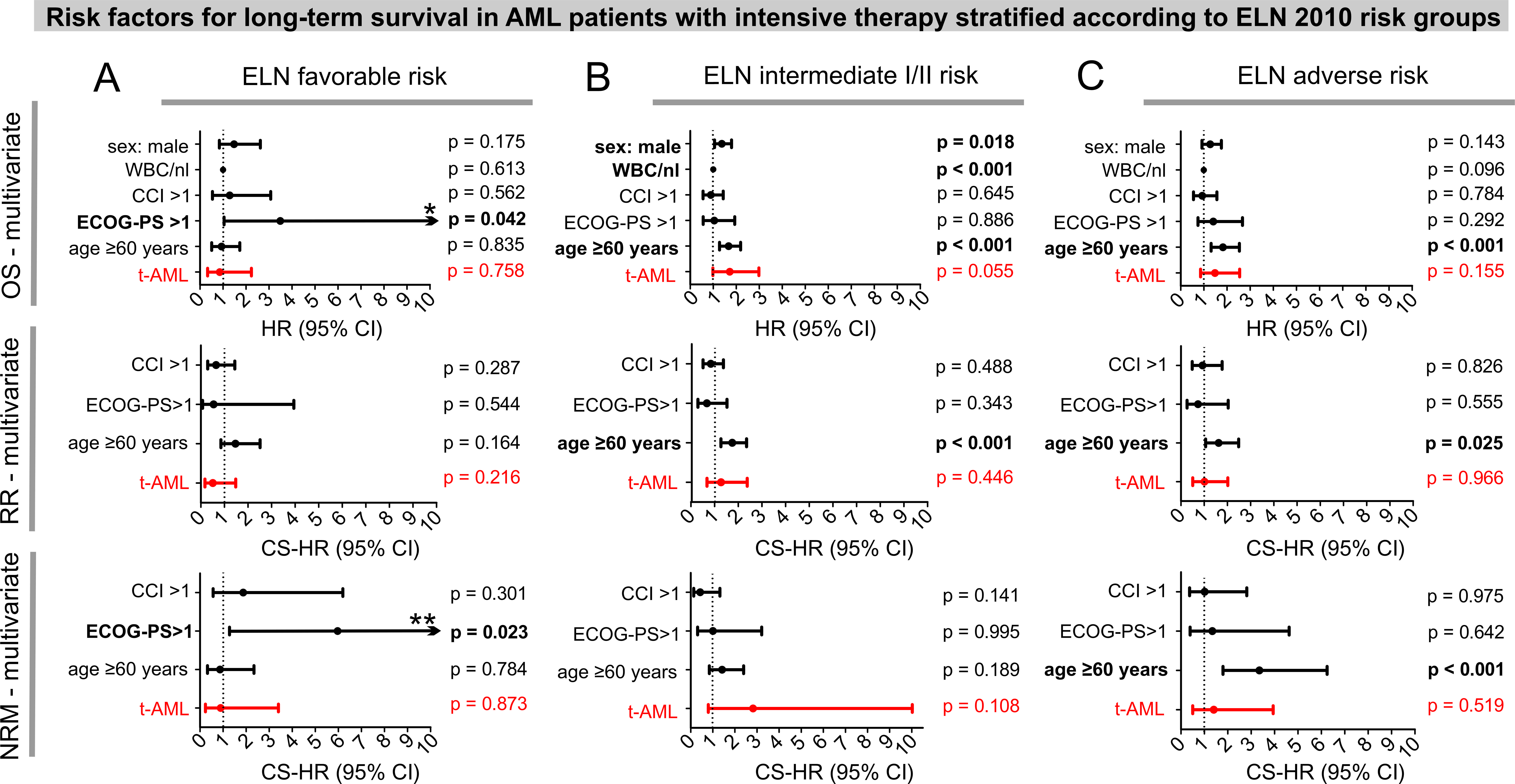

Supplement: Supplementary file 1 — Supplemental Figure 1 [file 41408_2024_1140_MOESM1_ESM.jpg]
