# Supplementary material for: Therapy-related AML: long-term outcome in a large cohort of AML-patients with intensive and non-intensive therapy
Source: Blood Cancer J. 2024 Sep 16;14(1):160. doi: 10.1038/s41408-024-01140-5 (PMC11405931; doi:10.1038/s41408-024-01140-5)
Supplement: Supplementary file 2 — Supplemental Figure 1 Legend [file 41408_2024_1140_MOESM2_ESM.docx]

**SUPPLEMENTAL FIGURE LEGEND**

**Supplemental Figure 1:** Multivariate analysis of risk factors for OS, RR and NRM in AML patients with intensive therapy as stratified according to ELN 2010. **A:** ELN favorable risk group (n=168). **B:** ELN intermediate I/II risk group (n =478) **C:** ELN adverse risk group (n=237).

**Abbreviations:** overall survival (OS), risk of relapse (RR), non-relapse mortality (NRM), therapy-related AML (t-AML), Charlson comorbidity index (CCI), ECOG performance score (ECOG), European LeukemiaNet (ELN), hazard ratio (HR), cause-specific hazard ratio (CS-HR), confidence interval (CI)

**Annotations:** *upper limit of 95% CI = 11.60, **upper limit of 95-% CI = 27.86
